# Comparative Evaluation of Near-Term Oncologic, Urinary, Sexual, and Postoperative Outcomes in Rectal Cancer: Laparoscopic vs. Robotic Approaches

**DOI:** 10.3390/medicina61101726

**Published:** 2025-09-23

**Authors:** Vagif Gurbanov, Veysel Umman, Osman Bozbiyik, Tayfun Yoldas

**Affiliations:** 1 Department of General Surgery and Organ Transplantation, Yeni Klinika Hospital, 142 Heydar Aliyev Avenue, Baku AZ1029, Azerbaijan; gurbanovagif@gmail.com; 2 Division of Organ Transplantation and Hepatopancreaticobiliary Surgery, Department of Surgery, Yeditepe Koşuyolu Hospital, Yeditepe University, 34718 İstanbul, Türkiye; veyselumman@gmail.com; 3 Department of General Surgery, Faculty of Medicine, Ege University, 35040 İzmir, Türkiye; bozbiyiko@gmail.com; 4 Department of General Surgery, Acıbadem Kent Hospital, 35620 İzmir, Türkiye

**Keywords:** laparoscopic, robotic, oncologic, rectum cancer, urinary, sexual, outcomes

## Abstract

*Background and Objectives:* This study compares laparoscopic and robotic surgical techniques for rectal cancer, focusing on oncologic outcomes, mesocolic excision quality, lymph node yield, and postoperative sexual and urinary function, while also exploring patient satisfaction and recovery trajectories through clinical outcomes and validated questionnaires. *Materials and Methods:* A retrospective analysis was conducted on 100 patients who underwent rectal cancer surgery between 2017 and 2021 at our tertiary center—53 underwent laparoscopic and 47 robotic surgery. Demographic data, tumor characteristics, and surgical details (procedure type, lymph node yield, morbidity, and mortality) were collected, and postoperative outcomes, including local recurrence, metastasis, need for reoperation, urinary incontinence, and sexual dysfunction, were compared. Functional outcomes were evaluated using the LARS questionnaire, Wexner score, IPSS, IIEF, and FSFI. *Results:* No significant differences were found in age, BMI, tumor size, or ASA scores between groups. Robotic surgery was associated with shorter hospital stays (*p* < 0.001), no conversions to open surgery (vs. 28.3% in laparoscopy), and zero cases of positive circumferential margins (vs. 35.8% in laparoscopy; *p* < 0.001). Lymphatic and perineural invasion rates were similar. Tumor recurrence occurred in four robotic and six laparoscopic cases, and factors significantly associated with recurrence included pathological stage, hospital stay, and adjuvant treatment. Robotic surgery showed improved urinary and sexual function, with lower Wexner, IPSS, and FSFI scores. *Conclusions:* Robotic surgery is a safe, effective, and patient-friendly alternative to laparoscopy, offering better preservation of continence and sexual function, reduced conversion rates, and shorter hospitalization, and should be considered the preferred approach in appropriately selected rectal cancer patients.

## 1. Introduction

The appropriate treatment modality for rectal cancer has been demonstrated to be highly effective in reducing the incidence of local recurrence and increasing survival rates. The etiology of local recurrence is multifactorial, involving lymphatic invasion and tumor positivity in the circumferential resection margin (CRM), with documented recurrence rates ranging from 3% to 32% [1,2,3]. Current surgical treatment involves en bloc removal of the rectum with negative margins proximally and distally, as well as removal of the mesorectum and draining lymph nodes, in accordance with the principles of total mesorectal excision (TME). Open, laparoscopic, and robotic surgical methods have been employed in the surgical management of rectal cancer. In recent years, there has been a noted shift toward minimally invasive techniques, which offer a series of advantages, including decreased hospital stay and recovery time, as well as the ability for patients to resume daily activities more rapidly, particularly in cases involving rectal cancer and narrow pelvic floor anatomy. Despite the fact that laparoscopic and robotic surgery are not universally appropriate for all patients due to patient-related factors and cost considerations, they have been shown to exhibit significant superiority to open surgery in the postoperative period [4].

Conventional laparoscopy introduced important benefits compared with open surgery—reduced wound complications, faster recovery and shorter hospital stay—but it has recognized technical challenges in the narrow male pelvis or in obese patients that can increase conversion rates or compromise the quality of the mesorectal plane. Robot-assisted platforms were introduced to address some of these limitations by offering articulated instruments, three-dimensional vision and improved ergonomics that may facilitate precise pelvic dissection. Randomized trials and comparative series to date have produced mixed results. Randomized data (ROLARR) showed no statistically significant reduction in the conversion rate for robotic versus laparoscopic TME but suggested potential surgeon-level benefits; subsequent meta-analyses and observational series report mixed results, with some studies showing improved preservation of urinary and sexual function after robotic TME and others showing comparable oncologic outcomes between techniques [5,6,7]. Given the ongoing debate regarding CRM, conversion, functional preservation, and cost, direct comparative series remain valuable to inform patient selection and service planning. The optimal minimally invasive approach to achieving high-quality TME with minimal morbidity is still under active investigation. At the same time, preservation of urinary, sexual, and anorectal function has become a parallel priority as survival improves; therefore, surgical approaches are now judged not only by immediate perioperative metrics and oncologic safety but also by their impact on quality of life.

With the accumulation of expertise, the challenges associated with minimally invasive surgery have been resolved, leading to its widespread acceptance. However, beyond conversion and oncologic metrics, functional outcomes—including bowel function (LARS), fecal incontinence (Wexner), urinary symptoms (IPSS) and sexual function (IIEF/FSFI)—are increasingly reported and influence patient counseling and shared decision-making. These outcomes are sensitive to patient factors, the extent of mesorectal dissection, autonomic nerve preservation, and the anastomotic technique, and they frequently require repeated, standardized assessments over time to capture recovery trajectories. In this context, we performed a retrospective single-center analysis comparing laparoscopic and robot-assisted rectal cancer resections. Our objective was to evaluate and contrast perioperative metrics (operative time, conversion, complications, length of stay), oncologic outcomes (CRM status, lymph node yield, recurrence and survival) and patient-reported functional outcomes (LARS, Wexner, IPSS, IIEF, FSFI) in order to contribute contemporary, practice-based evidence to inform patient selection and institutional planning.

We hypothesized that robotic TME would be associated with lower conversion rates and at least comparable oncologic outcomes compared with conventional laparoscopy, and that robotic dissection might offer advantages in early preservation of urinary and sexual function in selected patients.

## 2. Materials and Methods

A retrospective analysis was conducted on 100 patients who underwent either laparoscopic or robotic surgery for rectal adenocarcinoma at our center between March 2017 and 2021.

Inclusion criteria were as follows: age ≥ 18 years, histologically confirmed primary rectal adenocarcinoma undergoing elective curative-intent resection at our center between March 2017 and December 2021, absence of synchronous distant metastases on preoperative imaging, and availability of operative and pathology reports.

Exclusion criteria were as follows: emergency or semi-emergency operations for bleeding or obstruction, recurrent rectal cancer following prior radical rectal surgery, palliative resections for stage IV disease, and insufficient clinical records.

All procedures followed institutional TME standards. Operations were performed by the same surgical team who are colorectal surgeons experienced in minimally invasive rectal surgery; technique differences (e.g., high vs. low ligation and lateral pelvic node dissection) were applied according to tumor stage and preoperative MRI findings. The central dissection plane aimed to follow TME principles in all cases; anastomotic technique (stapled vs. hand-sewn), stoma policy (protective ileostomy for low rectal anastomoses), and the ERAS perioperative pathway were consistent.

A comprehensive review of medical records, demographic data, operative reports, pathology findings, and imaging results was performed. Tumor distance from the anal verge was measured on preoperative MRI and endoscopy.

Patients were categorized into two groups based on the surgical approach: laparoscopic and robotic. “Laparoscopic” indicates conventional multi-port laparoscopic TME performed without robotic assistance. “Robotic” indicates procedures performed with the da Vinci platform, with recorded docking and console times. Surgical approach (laparoscopic vs. robotic) was selected by the operating surgeon based on tumor characteristics, pelvic anatomy, and availability of the robotic platform; patient preference was considered when feasible. Demographic and clinical variables, including age, gender, comorbidities, tumor location, and type of surgery (Low Anterior Resection [LAR], Ultra Low Anterior Resection [Ultra LAR], and Abdominoperineal Resection [APR]), were analyzed. Total operative time (skin-to-skin), docking and console times (robotic cases), estimated blood loss, conversion to open, creation of stoma type, and intra-operative complications were analyzed. Postoperative outcomes such as length of hospital stay, morbidity (assessed using Clavien–Dindo classification), mortality, and tumor distance from the surgical margin were also evaluated. Pathology variables included tumor size, pTNM stage, number of harvested lymph nodes, lymphovascular and perineural invasion, and CRM status (positive defined as ≤1 mm).

The study further compared the postoperative adjuvant treatment, local recurrence, distant metastasis, need for reoperation, hospital readmission, urinary incontinence, and sexual function between the two groups. Additionally, functional outcomes were assessed using validated questionnaires, including the Low Anterior Resection Syndrome (LARS) score, Wexner score, International Prostate Symptom Score (IPSS), International Index of Erectile Function (IIEF), and Female Sexual Function Inventory (FSFI). Functional outcomes were assessed using these validated questionnaires (LARS, Wexner, IPSS, IIEF, FSFI) administered preoperatively when available and at clinic follow-up visits. Our institutional follow-up schedule aimed for postoperative assessment at approximately 3, 6, and 12 months; due to missing follow-up or incomplete questionnaire return, not all patients completed each time point. Given incomplete repeated measures, functional data are presented principally as cross-sectional comparisons and exploratory analyses. Because multiple exploratory comparisons were performed (particularly among functional endpoints), we did not apply a formal multiplicity correction; findings for these endpoints should therefore be considered hypothesis generating and interpreted in the context of a potential type-I error.

A comprehensive statistical analysis was conducted to evaluate immediate postoperative oncological and functional outcomes in patients undergoing laparoscopic and robotic rectal surgery.

Statistical analyses were conducted using SPSS v25.0 software. For numerical variables, the mean ± standard deviation was employed, while percentages and numerical data were utilized for qualitative variables. The normality of within-group distribution between cross groups was assessed using the Shapiro–Wilk test. Independent data sets exhibiting normal distribution were subjected to Student’s *t*-test, while dependent data sets were evaluated using Paired *t*-test and ANOVA test. For data sets failing to demonstrate normal distribution, Mann–Whitney U and Kruskall–Wallis tests were employed, contingent upon the number of groups. Survival analysis was performed using the Kaplan–Meier method. The chi-square test was used to evaluate categorical data. The confidence interval was set at 95%, and a *p*-value less than 0.05 was considered significant.

A post hoc power assessment indicated that the sample size in this single-center series provides limited power to detect small differences in recurrence or survival between groups. For example, to detect a difference in recurrence rates similar to our observed proportions (≈8.5% vs. 11.3%) with 80% power and α = 0.05 would require ~1800 patients per arm— far larger than our cohort—so negative findings for recurrence should be interpreted as exploratory. Thus, oncological outcome comparisons are presented descriptively and interpreted cautiously.

Statistical analyses were performed using Fisher’s exact test for categorical variables with small expected cell counts, and multivariable logistic regression was applied as an adjusted sensitivity analysis. Follow-up time is presented as mean ± standard deviation and median (range). Although time-to-event modeling with Cox proportional hazards regression was initially considered, the small number of recurrence events (*n* = 10) and the marked difference in follow-up duration between laparoscopic and robotic groups (mean 30.7 vs. 12.8 months) resulted in unstable estimates that would have been difficult to interpret. For this reason, recurrence was instead analyzed with multivariable logistic regression, adjusting for surgical approach, age, sex, and pathologic stage, to provide a conservative and reliable assessment. For comparisons of circumferential resection margin (CRM) positivity, Fisher’s exact test was used; adjusted logistic regression was not feasible because of complete separation (no CRM-positive cases in the robotic group). Odds ratios (ORs) with 95% confidence intervals (CIs) are reported, and two-sided *p*-values < 0.05 were considered statistically significant.

The study was approved by the Medical Research Ethics Committee of our university (Decision number: 23-6T/49, Date: 12 June 2023). Informed consent was obtained from each patient included in the study.

## 3. Results

In the present study, 100 patients who underwent surgery for rectal cancer were analyzed, with 47 patients undergoing robotic surgery and 53 patients undergoing laparoscopic surgery.

The mean age of patients in the robotic group was 58.09 (±13.83) years, while in the laparoscopic group it was 60.55 (±11.18) years, with no statistically significant difference observed between the two groups. The mean body mass index (BMI) was 27.47 (±3.89) in the robotic group and 27.01 (±3.73) in the laparoscopic group, with no significant difference noted. Tumor size also showed no statistically significant difference between the groups, with a mean of 4.21 cm (±2.34) in the robotic group and 4.68 cm (±2.27) in the laparoscopic group.

However, a statistically significant difference was observed in both the duration of hospitalization and follow-up period between the robotic and laparoscopic surgery groups (*p* < 0.05). The mean follow-up period was 12.77 ± 9.27 months (robotic) vs. 30.68 ± 17.79 months (laparoscopic); the mean difference was −17.91 months (95% CI −23.47 to −12.35 months). The mean length of hospitalization was 7.28 ± 1.86 days (robotic) vs. 10.23 ± 6.80 days (laparoscopy); and the mean difference was −2.95 days (95% CI −4.90 to −1.00 days). A detailed summary of the demographic characteristics and their respective *p*-values is presented in Table 1. Where statistically significant differences existed (for example, the longer follow-up time and higher proportion with the advanced pathological stage in the laparoscopic group), we highlight these and discuss their likely impact on recurrence and other outcomes in the discussion. Given these imbalances, we interpret between-group outcome comparisons cautiously.

The mean docking time was 12.26 min (±4.64), and the mean console time was 124.74 min (±27.04). The mean laparoscopic surgery time was 163.89 min (±45.51).

In the robotic surgery group, 21 patients (44.7%) were women and 26 (55.3%) were men, while in the laparoscopic group, 18 patients (34.0%) were women and 35 (66.0%) were men. An analysis of comorbidities showed that 30 patients (63.8%) in the robotic group and 36 patients (67.9%) in the laparoscopic group had a history of comorbid conditions. Additionally, a family history of rectal cancer was documented in five patients (10.6%) in the robotic group and four patients (7.5%) in the laparoscopic group.

The distribution of ASA (American Society of Anesthesiologists) scores was as follows: in the robotic group, 22 patients (46.8%) had an ASA score of 1, 21 patients (44.7%) had an ASA score of 2, 3 patients (6.4%) had an ASA score of 3, and 1 patient (2.1%) had an ASA score of 4. In the laparoscopic group, 18 patients (34.0%) had an ASA score of 1, 33 patients (62.3%) had an ASA score of 2, 1 patient (1.9%) had an ASA score of 3, and 1 patient (1.9%) had an ASA score of 4. There was no statistically significant difference between the two groups in terms of ASA scores (Table 2).

Regarding lymph node involvement, 21 patients (44.7%) in the robotic group and 39 patients (73.6%) in the laparoscopic group had lymph node positivity, a difference that was statistically significant (*p* < 0.001).

Imaging analysis revealed that 26 patients (55.3%) scheduled for robotic surgery and 30 patients (56.6%) scheduled for laparoscopic surgery underwent PET scans. However, a statistically significant difference was observed between the two groups (*p* = 0.003). Additionally, MRI imaging was performed in 23 patients in the robotic group and 41 patients in the laparoscopic group.

Regarding neoadjuvant chemotherapy, 35 patients (74.5%) in the robotic surgery group received treatment, compared to only 8 patients (17.0%) in the laparoscopic group. This difference was statistically significant.

An analysis of surgical procedures revealed that robotic LAR was performed in 35 patients (74.5%), robotic Ultra LAR in 8 patients (17.0%), and robotic APR in 4 patients (7.5%). In the laparoscopic group, LAR was performed in 49 patients (92.5%) and Ultra LAR in 4 patients (7.5%). Statistical analysis showed no significant difference between the distribution of operation types within the respective groups.

Stoma formation was observed in 22 patients (47.8%) in the robotic group and 21 patients (39.6%) in the laparoscopic group. In all robotic cases, the stoma type was a protective ileostomy.

Regarding conversion to open surgery, conversion occurred in 0/47 (0.0%; 95% CI for proportion 0.0–7.6%) robotic cases versus 15/53 (28.3%; 95% CI 17.97–41.6%) laparoscopic cases. The absolute difference in conversion rate was −28.3% (95% CI −40.4% to −16.2%). This difference was statistically significant (*p* < 0.001).

Postoperative complications were observed in one patient (2.2%) in the robotic group and two patients (3.8%) in the laparoscopic group. In the robotic group, one patient developed postoperative ileus, which resolved with conservative management, allowing for discharge without surgical intervention. In contrast, two patients in the laparoscopic group experienced anastomotic leakage, both of whom required colostomy formation.

In the robotic group, two patients (4.3%) experienced mortality, whereas no deaths were reported in the laparoscopic group. Neither of the robotic deaths was an immediate operative mortality. Among the deceased patients in the robotic group, one was lost due to local recurrence and metastatic disease in the fourth postoperative month following discharge, while the other passed away in the tenth postoperative month due to cardiac complications.

A statistically significant difference was observed in the distribution of pathological stages between the robotic and laparoscopic groups (*p* < 0.001). The stage distribution for each group is presented in Table 2. Pathological lymph node involvement was identified in 10 patients (21.3%) in the robotic group and 18 patients (34%) in the laparoscopic group.

Notably, none of the patients in the robotic group had a positive circumferential margin, 0/47 (0.0%; 95% CI 0.0–7.6%). Whereas 19/53 cases in the laparoscopic group (35.8%; 95% CI 24.3–49.3%) exhibited circumferential margin positivity, the absolute difference was −35.8% (95% CI −48.8% to −22.9%), a finding that was statistically significant (Fisher’s exact *p* < 0.001).

Regarding invasion characteristics, lymphatic and perineural invasion was detected in 10 patients (21.3%) in the robotic group and 12 patients (22.6%) in the laparoscopic group. No statistically significant difference was found between the two groups in terms of invasion patterns. Adjuvant oncologic treatment was administered to 30 patients (63.8%) in the robotic group and 36 patients (67.9%) in the laparoscopic group.

Regarding recurrence rates, recurrence occurred in 4/47 (8.5%; 95% CI 3.4–19.9%) robotic vs. 6/53 (11.3%; 95% CI 5.3–22.6%) laparoscopic patients; absolute difference is −2.8% (95% CI −14.5% to +8.9%) (Fisher’s exact *p* = 0.7457). However, the difference between the groups was not statistically significant.

When the factors contributing to recurrence in patients with postoperative recurrence were individually analyzed, it was found that the presence of a stoma, the type of surgery (robotic or laparoscopic), the surgical technique performed, and the ASA score had no statistically significant impact on recurrence. However, the pathological stage was found to have a statistically significant effect on recurrence (*p* < 0.001) (Table 3).

Continuous factors that could influence recurrence were statistically analyzed using the *t*-test. The analysis revealed that age, body mass index, tumor size, laparoscopic surgery duration, and total operation time had no statistically significant impact on recurrence. However, the length of hospitalization was found to have a statistically significant effect on recurrence (*p* < 0.001) (Table 4).

The remaining parameters in patients with recurrence were evaluated using the chi-square test. The analysis showed that gender, presence of comorbidities, family history, lymph node positivity, stoma creation, conversion to open surgery, complications, mortality, pathological lymph node involvement, circumferential margin positivity, and presence of lymphatic or perineural invasion did not have a statistically significant impact on recurrence (Table 5). However, a statistically significant increase in recurrence rate was observed in patients who received adjuvant treatment, underwent PET imaging, and received neoadjuvant treatment (Table 5).

Patients were invited to participate in outpatient clinic follow-ups, during which they were assessed using questionnaires to determine the LARS score and the Wexner score. Male patients were evaluated using the IPSS and the IIEF, while female patients were assessed using the FSFI. Due to the inadequate sample size for statistical analysis, no statistically significant results were obtained.

The mean Wexner score was found to be 7.69 ± 2.16. Among female patients, the mean FSFI score was 10.19 ± 8.64. In male patients, the mean IPSS score was 6.18 ± 1.50, while the mean IIEF score was 14.95 ± 5.33.

In a multivariable logistic regression model adjusting for age, sex and pathologic stage, pathologic stage was independently associated with recurrence (adjusted OR 2.83, 95% CI 1.16–6.92; *p* = 0.022). The adjusted odds ratio for robotic versus laparoscopic approach was 1.29 (95% CI 0.30–5.52; *p* = 0.73). Because CRM positivity was absent in the robotic arm (complete separation), adjusted logistic regression for CRM could not be performed; the unadjusted association remained highly significant. Given the low number of recurrence events and the unequal follow-up, these adjusted analyses should be interpreted as exploratory (Table 6).

## 4. Discussion

Rectal cancer is a prevalent disease with a high mortality rate. Advancements in technology, in conjunction with the emergence of novel diagnostic and therapeutic modalities, have led to improvements in cure rates and overall survival. Due to the superiority of minimally invasive techniques, these have become increasingly popular.

The evolution of laparoscopy and the increasing utilization of robotic surgery in rectal adenocarcinomas have led to the observation of variations in outcomes between these two surgical modalities. In this study, we conducted a retrospective analysis of patients who underwent surgical intervention, deriving several findings.

The study population comprised 100 patients who underwent surgical intervention for rectal cancer, with 47 undergoing robotic surgery and 53 undergoing laparoscopic surgery. A statistically significant finding was observed in the mean length of hospital stay, where mean hospital stay was 7.28 ± 1.86 days (robotic) vs. 10.23 ± 6.80 days (laparoscopy); the mean difference was −2.95 days (95% CI −4.90 to −1.00 days). This finding aligns with the results of a meta-analysis conducted by Flynn et al., which also reported that patients undergoing robotic surgery had a significantly shorter hospital stay compared to those who underwent laparoscopic surgery [8]. The hospital stay durations observed in our study for both surgical techniques are consistent with the literature.

A study conducted in 2020 reported that lateral lymph node dissection was more frequently performed in laparoscopic and robotic rectal surgery. Robotic cases were found to provide greater comfort and confidence for the surgeon compared to laparoscopic cases [9]. A similar study found no significant difference in pathological outcomes [10]. In the present study, lymph node involvement was detected in 73.6% of laparoscopic cases, whereas it was observed in 44.7% of robotic cases. Laparoscopic surgery was preferred for patients with a higher number of lymph nodes detected in preoperative imaging, and the difference between the two groups was found to be statistically significant. While our results do not contradict the existing literature, they do appear to support a greater tendency for laparoscopic cases in lymph node dissection.

In our study, the conversion rate to open surgery was 28.3% in laparoscopic cases, whereas there were no conversions in robotic cases. This may be due to the fact that 77.4% of robotic cases were evaluated by MRI. A study from 2020 highlighted the importance of patient selection for robotic surgery and reported significantly lower conversion rates for robotic procedures compared to the laparoscopic approach [11].

A subsequent analysis of pathological staging between the two groups revealed that robotic cases were predominantly classified as Stages 1 and 2, while laparoscopic cases were more common in Stages 2 and 3. This finding suggests that laparoscopic surgery is preferred for more advanced rectal cancer cases, which may also explain the higher conversion rate in laparoscopic procedures. A study suggested the need to establish robotic surgery as the standard of care for low stage rectal adenocarcinoma [10]. The present findings are consistent with these recommendations in the published literature.

The rate of positive circumferential margin was 35.8% in the laparoscopic group, whereas no cases were detected in the robotic group. This outcome is believed to be influenced by the preference for laparoscopic surgery in higher-stage and more complex cases. Patients with metastatic lymph node involvement, conversion to open surgery, advanced pTNM stage, positive circumferential margin, and recurrence during follow-up were more frequently observed in the laparoscopic group. This outcome is associated with more advanced disease. Robotic surgery is widely regarded as the preferred approach for low-stage rectal cancer, a preference that is well-documented in the published literature.

The recurrence rates were also analyzed during follow-up, revealing a correlation between higher pathological stage and increased recurrence rates. A previous study similarly reported that as tumor grade increases, the likelihood of recurrence during follow-up also rises [12].

In our study, recurrence was observed more frequently in patients who received neoadjuvant and adjuvant therapy than in those who did not experience recurrence during follow-up, and this difference was found to be statistically significant. Given that decisions regarding neoadjuvant and adjuvant therapy are made according to the AJCC staging system, this result is considered to be in accordance with expectations.

The assessment of LARS, erectile dysfunction, female sexual dysfunction, and urinary functions during follow-up involved the utilization of the Wexner score, FSFI score, IPSS, and IIEF score. Despite the inability to conduct a comprehensive statistical evaluation due to the limited availability of data, the obtained results exhibited similarities to those reported by Flynn J. et al. in their 2022 study. The study by Flynn et al. also indicated that robotic surgery yielded superior outcomes. Our findings are in line with the study by Li et al. and also recent systematic review and meta-analysis by Zhu et al. as it was shown that compared with laparoscopic, robotic TME had better preservation of urinary and sexual functions with comparable long term oncological outcomes in rectal cancer [6,13]. We emphasize that functional outcomes were collected in a subset of patients and therefore the findings should be considered exploratory. Psychosexual and broader quality-of-life instruments were not included in our routine follow-up; we explicitly acknowledge this as a gap and recommend future studies integrate validated quality of life measures While our findings align with the existing literature in this regard, further studies with larger datasets are necessary to strengthen the conclusions.

A comparative analysis of laparoscopic and robotic cases revealed no statistically significant disparities between the two groups with respect to age, sex, comorbidities, BMI, family history, ASA scores, tumor size, complications during follow-up, mortality, or the receipt of neoadjuvant and adjuvant therapy.

In the current study, the mean length of hospital stay was 7.28 (±1.8) days in the robotic group. In contrast, the laparoscopic group exhibited a mean length of stay of 10.23 (±6.8) days, a difference that was found to be statistically significant (*p* < 0.001). This finding aligns with the existing literature on the subject [8].

The findings in our study are directly related to the higher disease stage in the laparoscopic group and are consistent with previous studies [10,11,12,14]. LARS and Wexner scores were assessed using questionnaires. Male patients were evaluated using the IPSS and the IIEF, while female patients were assessed using the FSFI. Despite the limited number of cases, the findings were consistent with the existing literature [9].

Our study findings further support the advantages of robotic surgery. In addition to no conversion to open surgery in the robotic group and no cases of CRM positivity in the robotic group, the mean hospital stay was also significantly shorter in the robotic group (7.28 ± 1.8 days) compared to the laparoscopic group (10.23 ± 6.8 days, *p* < 0.001). Lymph node involvement was also lower in robotic cases (44.7%) compared to laparoscopic cases (73.6%), a difference that likely reflects the greater proportion of advanced-stage tumors treated laparoscopically in our cohort.

This study offers several strengths that enhance its clinical and scientific relevance. It provides a comprehensive, real-world comparison of robotic and laparoscopic approaches to rectal cancer surgery, integrating both oncologic and functional outcomes. The inclusion of validated assessment tools—such as the Wexner score, IPSS, IIEF, and FSFI—adds depth to the evaluation of postoperative quality of life, a domain that remains underreported in the literature. Furthermore, the study includes an analysis of recurrence patterns in relation to surgical technique, adjuvant therapy, and pathological staging, offering valuable prognostic insights.

Cost and operating time are important considerations. Robotic procedures frequently incur higher direct device cost and longer operation durations (docking and console time), but may reduce conversion rates, length of stay, and postoperative complication–related costs. The net economic impact therefore depends on institution caseload, amortization, and integration with Enhanced Recovery After Surgery (ERAS) pathways. Several centers report a greater per-case cost for robotic surgery that may be partially offset by shorter hospital stays and lower conversion-related costs; however, randomized evidence for cost-effectiveness is limited and context dependent. In this series we present operative times and length of stay to support local health-economics appraisal; a formal cost-effectiveness analysis is beyond the scope of the present paper.

Robotic surgery is increasingly recommended for mid-to-low rectal tumors in centers with appropriate surgical expertise. Its technical advantages—including enhanced visualization, greater dexterity, and improved ergonomics—may contribute to superior functional outcomes and more precise surgical dissection, particularly in narrow pelvic anatomy. Additionally, robotic surgery facilitates more effective lymph node dissection, which may contribute to improved oncologic control. Although concerns regarding cost persist, emerging data suggest that when robotic surgery is combined with ERAS protocols and appropriate case selection, it may offer cost-effective benefits. However, in complex and advanced-stage rectal cancers, laparoscopic surgery continues to be the preferred approach in many centers, underscoring the importance of MRI-based preoperative staging to guide individualized surgical planning.

Our results both align with and diverge from prior evidence. The largest randomized controlled trial to date, ROLARR, reported that robotic surgery did not significantly reduce the conversion rate to open surgery compared with laparoscopy; conversion was 8.1% in the robotic group and 12.2% in the laparoscopic group (adjusted OR 0.61; 95% CI 0.31–1.21; *p* = 0.16). This finding suggests that robotics may not confer a universal technical advantage [5].

Functional outcomes remain an area of ongoing debate. A recent meta-analysis demonstrated that robotic surgery was associated with improved urinary and erectile function recovery in men, with lower IPSS and better IIEF scores compared to laparoscopy at six months (IPSS mean difference −1.08; 95% CI −1.85 to −0.30; *p* = 0.007) [15]. Another systematic review from 2024 also emphasized that robotics may provide better preservation of urinary and sexual function in both men and women [6]. By contrast, other pooled analyses suggest that functional outcomes after total mesorectal excision (TME) are generally comparable between laparoscopic and robotic approaches, with only a modest short-term advantage of robotics for urinary function at three months (SMD −0.15; 95% CI −0.24 to −0.06; *p* = 0.02) [16].

In our series, robotic cases demonstrated more favorable trends in urinary and sexual function scores. However, the smaller sample size and incomplete follow-up limit the strength of these findings. Taken together, our study supports the possibility of functional benefits with robotics, but also underscores the need for larger, prospective, multicenter studies with standardized functional outcome assessments to confirm these observations.

Despite these strengths, several limitations must be acknowledged. This is a retrospective observational study of consecutive elective rectal cancer resections from 2017 to 2021. The retrospective, single-center design introduces the potential for selection bias and may limit the generalizability of the findings. Baseline imbalances (notably more advanced pathological stage and longer follow-up in the laparoscopic group) confound some between-group comparisons and may bias recurrence and CRM outcomes. The sample size limits statistical power to detect small oncologic differences and constrains multivariable modeling for some endpoints. Although we compared a broad set of perioperative, oncologic, and functional endpoints, the limited number of oncologic events (10 recurrences) prevented robust multivariable modeling: the commonly used rule of thumb of ≥10 events per covariate would have been violated, leading to overfitting and unreliable adjusted hazard ratios. For the same reason, we did not perform propensity-score matching. We therefore report unadjusted results and advise interpreting oncologic comparisons (particularly recurrence and CRM) with caution because baseline imbalances (notably stage and follow-up duration) may bias these results. Cost and quality-of-life instruments beyond the sexual/urinary scores were not collected. Finally, learning-curve effects during the robotic adoption period may have influenced operative times and outcomes. We have emphasized these limitations and recommend prospective, propensity-matched or randomized multi-center studies for more definitive comparisons.

On the other hand, the retrospective design allowed for the capture of real-world practices and outcomes during the period when the robotic platform was introduced and integrated into our center’s workflow. While prospective randomized trials (e.g., ROLARR) provide the highest evidence, retrospective cohorts complement trial data by reflecting everyday patient selection, learning-curve effects, and resource constraints. The shorter median follow-up duration in the robotic group also poses a risk of underestimating late-onset complications or tumor recurrences. We acknowledge limitations inherent to retrospective studies and these factors highlight the need for prospective, multicenter studies with longer follow-up periods and balanced cohorts to validate and expand upon our findings.

## 5. Conclusions

In summary, in this single-center retrospective series, robotic rectal surgery was associated with shorter length of stay, lower conversion rates, and fewer positive CRM cases compared with laparoscopy. Functional scores in a limited subset suggested better urinary and sexual function preservation after robotic surgery but were exploratory because of small numbers. Nevertheless, laparoscopy remains a valid option, particularly in complex or advanced-stage cases and in centers without established robotic programs. Individualized, multidisciplinary patient selection remains essential. Given the baseline imbalances and limited event counts, oncologic conclusions must be cautious: our data suggest robotic surgery is a feasible and potentially advantageous approach in selected patients, but larger prospective and propensity-matched studies with longer follow-up are required to confirm whether these perioperative and functional advantages translate into durable oncologic benefit.

## Figures and Tables

**Table 1:** Demographic values in robotic and laparoscopic groups.

Values	Robotic	Laparoscopic		*p*-Value
	Mean (Std. Deviation)	Mean (Std. Deviation)		
**Age**	58.09 (±13.831)	60.55 (±11.18)		0.334
**BMI**	27.47 (±3.89)	27.01 (±3.73)		0.55
**Tumor size (cm)**	4.21 (±2.34)	4.68 (±2.27)		0.315
**Follow up time (mo)**	12.77 (±9.272)	30.68 (±17.79)		**<0.001**
**Hospitalization time**	7.28 (±1.860)	10.23 (±6.80)		**<0.001**
**Laparoscopic time**	163.89 (±45.51)			
**Docking time**	12.26 (±4.64)			
**Console time**	124.74 (±27.04)			
**Surgery duration (min)**	288.85 (±49.68)			

**Table 2:** Comparison between robotic and laparoscopic groups.

		Robotic	Laparoscopic	*p*-Value
		*n* (%)	*n* (%)	
**Gender**	Female	21 (44.7%)	18 (34.0%)	0.273
	Male	26 (55.3%)	35 (66.0%)	
**Comorbidities**	Present	30 (63.8%)	36 (67.9%)	0.668
	Absent	17 (36.2%)	49 (92.5%)	
**Family history**	Present	5 (10.6%)	4 (7.5%)	0.592
	Absent	42 (89.4%)	49 (92.5%)	
**ASA score**	1	22 (46.8%)	18 (34.0%)	0.364
	2	21 (44.7%)	33 (62.3%)	
	3	3 (6.4%)	1 (1.9%)	
	4	1 (2.1%)	1 (1.9%)	
**Lymph node**	Absent	26 (55.3%)	14 (26.4%)	<0.001
	Present	21 (44.7%)	39 (73.6%)	
**PET**	Absent	21 (44.7%)	23 (43.4%)	0.898
	Present	26 (55.3%)	30 (56.6%)	
**MRI**	Absent	24 (51.1%)	12 (22.6%)	<0.001
	Present	23 (48.9%)	41 (77.4%)	
**Neoadjuvant**	Absent	12 (25.5%)	18 (34.0%)	0.359
	Present	35 (74.5%)	35 (66.0%)	
**Robotic cases**	Robotic LAR	35 (74.5%)	49 (92.5%)	0.012
	Robotic Ultra LAR	8 (17.0%)	4 (7.5%)	
	Robotic APR	4 (8.5%)	0 (0.0%)	
**Stoma**	Absent	24 (52.2%)	32 (60.4%)	0.411
	Present	22 (47.8%)	21 (39.6%)	
**Stoma type**	0	24 (51.1%)	32 (60.4%)	0.54
	1	20 (42.6%)	15 (28.3%)	
	2	3 (6.4%)	6 (11.3%)	
**Conversion to open**	Absent	47 (100.0%)	38 (71.7%)	<0.001
	Present	0 (0.0%)	15 (28.3%)	
**Complication**	Absent	45 (97.8%)	51 (96.2%)	0.643
	Present	1 (2.2%)	2 (3.8%)	
**Closure of ileostomy**	Absent	41 (89.1%)	49 (92.5%)	0.566
	Present	5 (10.9%)	4 (7.5%)	
**Mortality**	Absent	44 (95.7%)	51 (100.0%)	0.132
	Present	2 (4.3%)	0 (0.0%)	
**Pathological grade**	0	5 (10.6%)	4 (7.5%)	<0.001
	1	19 (40.4%)	9 (17.0%)	
	2	16 (34.0%)	18 (34.0%)	
	3	6 (12.8%)	21 (39.6%)	
	4	1 (2.1%)	1 (1.9%)	
**Total pathological lymph node**	Absent	37 (78.7%)	35 (66.0%)	0.151
	Present	10 (21.3%)	18 (34.0%)	
**Pathological lymph node**	Absent	37 (78.7%)	35 (66.0%)	0.159
	Present	10 (21.3%)	18 (34.0%)	
**Pathological result**	Absent	42 (89.4%)	35 (66.0%)	<0.001
	Present	5 (10.6%)	18 (34.0%)	
**Surgical margin positivity**	Absent	47 (100.0%)	34 (64.2%)	<0.001
	Present	0 (0.0%)	19 (35.8%)	
**Invasion** **(Lymph. Perineural)**	Absent	37 (78.7%)	41 (77.4%)	0.869
	Present	10 (21.3%)	12 (22.6%)	
**Adjuvant treatment**	Absent	17 (36.2%)	17 (32.1%)	0.666
	Present	30 (63.8%)	36 (67.9%)	
**Recurrence**	Absent	43 (91.5%)	47 (88.7%)	0.640
	Present	4 (8.5%)	6 (11.3%)	

**Table 3:** Factors affecting recurrence.

		Recurrence		*p*-Value
		Absent	Present	
		*n* (%)	*n* (%)	
**Stoma**	Absent	53 (58.9%)	3 (30.0%)	0.117
	Ileostomy	29 (32.2%)	6 (60.0%)	
	Colostomy	8 (8.9%)	1 (10.0%)	
**Robotic**	Robotic LAR	76 (84.4%	8 (80.0%)	0.773
	Robotic Ultra LAR	10 (11.1%)	2 (20.0%)	
	Robotic APR	4 (4.4%)	0 (0.0%)	
**Pathologic grade**	0	9 (10.0%)	0 (0.0%)	<0.001
	1	27 (30.0%)	1 (10.0%)	
	2	31 (34.4%)	3 (30.0%)	
	3	22 (24.4%)	5 (50.0%)	
	4	1 (1.1%)	1 (10.0%)	
**ASA score**	1	37 (41.1%)	3 (30.0%)	0.447
	2	48 (53.3%)	6 (60.0%)	
	3	4 (4.4%)	0 (0.0%)	
	4	1 (1.1%)	1 (10.0%)	

**Table 4:** Continuous variable affecting recurrence.

Recurrence	*n*	Mean	Std. Deviation	*p*-Value
**Age**	90	59.37	13.034	0.922
	10	59.60	6.059	
**BMI**	90	27.0948	3.87569	0.200
	10	28.4070	2.79152	
**Tumor size (cm)**	90	4.4278	2.31818	0.672
	10	4.7500	2.25154	
**Laparoscopy duration**	43	163.86	43.809	0.972
	4	164.25	69.897	
**Console duration**	43	125.12	27.720	0.716
	4	120.75	20.710	
**Surgery duration (min)**	43	289.21	49.133	0.905
	4	285.00	63.509	
**Follow up duration (mo)**	90	22.54	17.416	0.516
	10	19.70	12.166	

**Table 5:** Other parameters affecting recurrence.

		Recurrence		*p*-Value
		Absent	Present	
		*n* (%)	*n* (%)	
**Study group**	Robotic	43 (47.8%)	4 (40.0%)	0.640
	Laparoscopic	47 (52.2%)	6 (60.0%)	
**Comorbidities**	Absent	31 (34.4%)	3 (30.0%)	0.778
	Present	59 (65.6%)	7 (70.0%)	
**Family history**	Absent	81 (90.0%)	10 (100.0%)	0.295
	Present	9 (10.0%)	0 (0.0%)	
**Lymph node**	Absent	37 (41.1%)	3 (30.0%)	0.496
	Present	53 (58.9%)	7 (70.0%)	
**PET**	Absent	44 (48.9%)	0 (0.0%)	<0.001
	Present	46 (51.1%)	10 (100.0%)	
**MRI**	Absent	33 (36.7%)	3 (30.0%)	0.677
	Present	57 (63.3%)	7 (70.0%)	
**Neoadjuvant**	Absent	30 (33.3%)	0 (0.0%)	<0.001
	Present	60 (66.7%)	10 (100.0%)	
**Stoma**	Absent	54 (60.0%)	2 (22.2%)	0.290
	Present	36 (40.0%)	7 (77.8%)	
**Conversion to open**	Absent	77 (85.6%)	8 (80.0%)	0.641
	Present	13 (14.4%)	2 (20.0%)	
**Complication**	Absent	86 (96.6%)	10 (100.0%)	0.555
	Present	3 (3.4%)	0 (0.0%)	
**Ileostomy closure**	Absent	82 (92.1%)	8 (80.0%)	0.206
	Present	7 (7.9%)	2 (20.0%)	
**Mortality**	Absent	86 (97.7%)	9 (100.0%)	0.648
	Present	2 (2.3%)	0 (0.0%)	
**Total pathological lymph node**	Absent	65 (72.2%)	7 (70.0%)	0.882
	Present	25 (27.8%)	3 (30.0%)	
**Metastatic** **Lymph Node**	Absent	70 (77.8%)	7 (70.0%)	0.578
	Present	20 (22.2%)	3 (30.0%)	
**Surgical margin positivity**	Absent	74 (82.2%)	7 (70.0%)	0.35
	Present	16 (17.8%)	3 (30.0%)	
**Adjuvant** **Treatment**	Absent	33 (36.7%)	1 (10.0%)	0.041
	Present	57 (63.3%)	9 (90.0%)	
**Invasion** **(Lymph. Perineural)**	Absent	71 (78.9%)	7 (70.0%)	0.520
	Present	19 (21.1%)	3 (30.0%)	
**Pathological lymph node group**	Absent	65 (72.2%)	7 (70.0%)	0.882
	Present	25 (27.8%)	3 (30.0%)	
**Pathology results**	Absent	70 (77.8%)	7 (70.0%)	0.579
	Present	20 (22.2%)	3 (30.0%)	
**Gender**	Female	37 (41.10%)	2 (20%)	0.192
	Male	53 (58.90%)	8 (80%)	

**Table 6:** Multivariable logistic regression for recurrence (sensitivity).

Predictor	Adjusted OR	95% CI	*p*-Value
**Group (robotic vs. laparoscopic)**	1.29	0.30–5.52	0.730
**Age (per year)**	1.01	0.96–1.07	0.675
**Sex (male vs. female)**	2.39	0.45–12.65	0.306
**Pathologic stage (per category increase)**	2.83	1.16–6.92	0.022

## Data Availability

The data presented in this study are available on request from the corresponding author. (Data is unavailable due to privacy or ethical restrictions.)

## Abbreviations

The following abbreviations are used in this manuscript:

CRM	Circumferential Resection Margin
TME	Total Mesorectal Excision
LAR	Low Anterior Resection
Ultra LAR	Ultra Low Anterior Resection
APR	Abdominoperineal Resection
LARS	Low Anterior Resection Syndrome
IPSS	International Prostate Symptom Score
IIEF	International Index of Erectile Function
FSFI	Female Sexual Function Inventory
BMI	Body Mass Index
ASA	American Society of Anesthesiologists
ERAS	Enhanced Recovery After Surgery
